# Optimization of an in vitro human blood–brain barrier model: Application to blood monocyte transmigration assays

**DOI:** 10.1016/j.mex.2015.11.009

**Published:** 2015-12-11

**Authors:** Alexandre Paradis, David Leblanc, Nancy Dumais

**Affiliations:** Département de Biologie, Faculté des Sciences, Université de Sherbrooke, Sherbrooke, QC, Canada J1 K 2R1

**Keywords:** Blood–brain barrier, Co-culture, Transmigration assays

## Abstract

The blood–brain barrier (BBB) is a selectively permeable barrier that separates the circulating blood from the extracellular fluid of the brain and is an essential component in brain homeostasis. In vitro BBB models are valuable supporting tools that can precede and complement animal and human studies of the development and progression of the central nervous system diseases. At present, mono-, co-, and tri-culture models that use porcine, murine, or human cells have been developed. We have optimized a two-dimensional model of the human BBB using primary human brain microvascular endothelial cells and normal human astrocytes. We have validated the effectiveness of our model with transmigration assays of human blood monocytes toward CCL19, a natural ligand of the chemokine receptor CCR7. This model offers the following advantages:

• It is simple, convenient, and requires small quantities of material, reagents, and primary cells.
• It can be used to monitor cell migration through the BBB.
• It can be used to assess brain capillary permeability in the presence of xenobiotic, pro-inflammatory, or other substances.

## Graphical abstract

**Figure:**
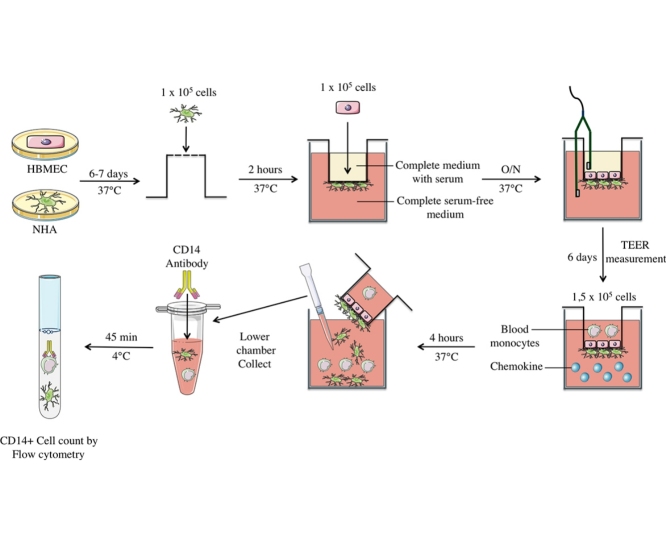

## Method details

### Introduction

Mimicking the physiology and functional responses of the blood–brain barrier (BBB) in vitro is a challenging task. Many techniques have been described including an in silico model, immobilized artificial membrane chromatography, and a parallel artificial membrane used for predicting drug permeability across specific physiological membranes in vivo [1], [2]. Over the years, new cell culture techniques and improved technologies have provided the necessary tools to create more realistic in vitro cell-based BBB models to advance our understanding of BBB physiology and function [1], [2], [3], [4]. Highly purified populations of cultured human brain cells (human brain microvascular endothelial cells [HBMEC]) and normal human astrocytes (NHA) exhibit excellent characteristics for studying the developmental and pathophysiological processes of the BBB. These cultures are more tedious to grow than other cell lines and require many technical skills to establish an appropriate BBB model. However, primary human cells used in co-culture provide a more realistic BBB model than mono-cultures and cell line co-cultures [1], [2], [3], [4].

Here, we describe a method based on the BBB model developed initially by Persidsky et al. and Ifergan et al. [3], [4], [5]. Primary human endothelial cells and astrocytes are co-cultured using Thincerts™ tissue culture inserts to obtain a selective and tight in vitro model of the human BBB. This method includes a detailed protocol to facilitate the generation of a functional in vitro BBB model optimized to perform transmigration assays of human blood monocytes in response to chemokines. The method is outlined below, in two steps.

### Step 1. Preparation of confluent human brain microvascular endothelial cells (HBMEC) and normal human astrocytes (NHA)

#### Materials and reagents

• Human Brain Microvascular Endothelial Cells (Cell Systems, Cat# ACBRI-376, Kirkland, WA, USA).
• Normal human astrocytes (Cell Systems, Cat# ACBRI-371).
• Complete Classic Medium Kit With Serum and CultureBoost (Cell Systems, Cat# C4Z0-500).
• CSC complete serum-free medium with RocketFuel (Cell Systems, Cat# SF-4Z0-500).
• “BAC-OFF” antibiotic (Cell Systems, Cat# 4Z0-643).
• Attachment Factors (Cell Systems, Cat# 4Z0-210).
• Passage Reagent Group™ (Cell Systems, Cat# 4Z0-800).
• Tissue culture dish, 150 mm (Life Technologies, Cat# 130183, Burlington, ON, Canada).
• PRIMARIA™ tissue culture dishes, 100 mm (Life Technologies, Cat# 353803).
• Cell scrapers (Sarstedt, Cat# 83.1830, Nümbrecht, NW, Germany).

#### Procedure

1. Preheat Attachment Factors at 37 °C.
2. Completely coat 100-mm adherent cell plates with 1.5 or 2.0 mL preheated Attachment Factors for HBMEC and NHA, respectively.
3. Thaw 7.5 × 10^5^ HBMEC (Passage 4) and 1 × 10^6^ NHA (Passage 4).
4. Culture the cells in the prepared plates in 10 mL Complete Classic Medium Kit with Serum plus antibiotics for the HBMEC, or 15 mL CSC complete serum-free medium with RocketFuel plus antibiotics for the NHA, according to the manufacturer's protocols.
5. Incubate the culture plates at 37 °C in 5% CO_2_.
6. Add fresh medium daily for 6 days. Under these experimental conditions, HBMEC and NHA should form a confluent monolayer and enough cells should be present at 6 days to form the model.
7. To form the human BBB co-culture model, gently remove HBMEC and NHA from their adherent plates by sharply rapping the culture vessel using cell scrapers with reagents from the Passage Reagent Group, according to the manufacturer's protocol.

### Step 2. Preparation of the human BBB co-culture model

#### Materials and reagents

• ThinCerts™ 3-μm tissue culture inserts for 24-well culture plates and assorted plates (Greiner Bio One, Cat # 662631, Monroe, NC, USA).
• 12-well culture plates (Corning, Cat # 662631, New York, NY, USA).
• Parafilm (Ultident, Cat # 662631, Saint-Laurent, QC, Canada).
• Fibronectin (Sigma–Aldrich, Cat# F1056-5MG, Oakville, ON, Canada).
• Ohm meter “EVOM” (World Precision Instrument, Cat# EVOM Sarasota, FL, USA).
• Chopstick Electrode Set for EVOM2, 4 mm (World Precision Instrument, Cat# STX2).
• 1× Phosphate-buffered saline (PBS; Wisent, Inc., Cat# 311-010-CL St-Bruno, QC, Canada).

#### Procedure

##### Description

**Fig. 1:**
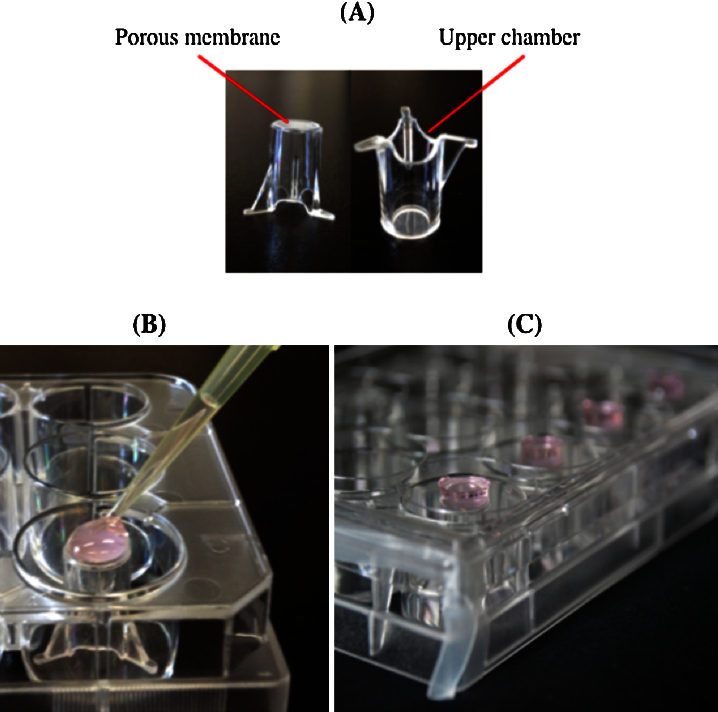
Method for NHA culture on the external side of membrane inserts. (A) Thincerts™ two-compartment system. Inserts positioned in a 12-well plate after the addition of NHA culture (B), and NHA culture adhesion between the insert and the 12-well plate cover (C).

The BBB model consists of two-compartment wells in a 24-well culture plate, with an upper and lower compartment separated by a ThinCert™ porous membrane. The membrane is a polyethylene terephthalate capillary pore membrane with a 3-μm pore size and a surface area of 0.336 cm^2^. This system mimics several aspects of the native in vivo conditions (Fig. 1A) and the membrane features a physical surface that provides for optimal cell attachment and growth, making the set-up ideal for study of the migration and invasion of cells through a selective membrane, such as the BBB. ThinCert™ is available with several membrane pore sizes and the best choice depends on the intended application. The 3-μm pore size used here is ideal for chemotactic migration and adds additional selectivity for monocyte transmigration, as monocytes are generally 15–25 μm in diameter.

##### Assessment of the BBB integrity

Confirmation of the BBB integrity is essential to perform reliable in vitro experiments. To do so, quantitative and qualitative techniques have been developed. Transendothelial Electrical Resistance (TEER) measurement has been extensively used to measure the resistance of tight junctions in BBB models [6], [7], [8], [9], [10], [11]. During model development, the cell monolayer on the apical membrane of the inserts becomes confluent. The tight junctions that form at this time will increase the TEER, which can be measured as an increase in electrical resistance. The resistance to ion flow across the BBB can be measured and used as a quantitative indicator of the “tightness” of the monolayer and cell health [1], [2], [3], [12]. The TEER value of HBMEC monolayers in vitro is estimated to be between 20 and 200 Ω cm^2^ [13]. The TEER value of a typical HBMEC-NHA co-culture is approximately 200 ± 50 Ω cm^2^ [14]. The TEER method is non invasive and is best suited to continuously monitor the BBB integrity in live cells during their various stages of growth and development [11]. Alternatively, the integrity of the in vitro human BBB model can be assessed by measuring the permeability of paracellular compounds of various molecular weights such as [14C] sucrose flux or Lucifer yellow permeability assays (reviewed in [11]). These tracer compounds indicate the endothelial permeability coefficient, the paracellular water flow as well as the pore size of the tight junctions [11], [15], [16], [17]. However, the use of such compounds renders the tested BBB unusable for further experiments. In addition, qualitative insights into the barrier integrity can be provided by immunostaining for proteins of tight junctions such as occluding, ZO-1 and ZO-2. Here, the barrier integrity of the BBB model was assessed by the noninvasive TEER method that is a strong indicator of the integrity of cellular barriers [11].

**Day 1:**

1. Add 50 μL of a 168 μg mL^−1^ solution of fibronectin in phosphate-buffered saline (PBS) to extend the external side of each membrane insert by 2.5 μg cm^2^.
2. Let dry for 3 h.
3. Dispose of the inserts in the 24-well plates.
4. Add 50 μL of a 168 μg mL^−1^ solution of fibronectin in PBS to extend the internal side of each membrane insert by 2.5 μg cm^2^.
5. Let dry overnight.

**Day 2:**

1. Preheat Attachment Factors and CSC complete serum-free medium (for NHA culture) at 37 °C.
2. Concentrate NHA and HBMEC to 1 × 10^7^ cells 20 μL^−1^ from a 5 × 10^4^ cells mL^−1^ preparation, according to the Passage Reagent Group protocol.
3. Place inverted inserts in 12-well plates.
4. Add 30 μL Attachment Factors on the external membrane insert and distribute evenly with a sterile pipet tip.
5. Once coated, add a band of sterile Parafilm to both sides of the plate so that it does not completely close later.
6. On the external sides of the membrane inserts containing the Attachment Factors, gently add 200 μL of NHA (Fig. 1B).
7. Gently put the lid of the 12-well plates containing inserts so that the plate is closed halfway, which will keep the NHA culture in contact with both the external membrane of the inserts and the lid (Fig. 1C). This step is critical. The NHA droplet must stay in contact with the lid and membrane insert during the 2-h incubation. The droplet will fall if the lid closes completely, and fewer astrocytes will lay down on the membrane, leading to incomplete co-culture and altered TEER values.
8. Incubate at 37 °C in 5% CO_2_ for 2 h.
9. During this time, fill each well that will contain inserts with 500 μL CSC complete serum-free medium for NHA culture.
10. After incubation, replace inserts with sterile pliers on the right side in the 24-well plate previously filled with NHA medium.
11. Add 50 μL Attachment Factors on the internal membrane insert surface of each insert.
12. Add 200 μL HBMEC.
13. Incubate at 37 °C in 5% CO_2_ for 2 h.
14. Add 100 μL Complete Classic Medium Kit with Serum for HBMEC culture into each insert.
15. Incubate overnight at 37 °C and 5% CO_2_.

**Day 3:**

1. Transfer each insert into a new 24-well plate previously filled with 500 μL of warmed CSC medium for NHA culture. This step prevents the presence of astrocytes in the lower chamber of the wells that might not have fully adhered on the external side of the membrane insert.
2. Gently remove HBMEC medium in each insert and add 300 μL of warmed medium for HBMEC culture; be careful not to pierce the membrane insert.
3. Measure TEER twice on each co-culture with an epithelial voltohmmeter after sterilizing the electrodes with 95% alcohol and rinsing with PBS. Measurements should be taken at room temperature 3–4 h after each medium renewal by placing the upper side of the electrode inside the inserts (HBMEC medium) without touching the membrane, and placing the lower side inside the well (NHA medium). TEER can vary dramatically depending on the method applied and precision during measurement. It is important to maintain the electrode distance and location when using chopsticks in different inserts, and to measure the resistance at least twice per insert to obtain reproducible results. To improve measurement reproducibility and stability, it is important that the electrodes stay immersed in the culture media and to maintain a steady position as excessive movement will cause measurement fluctuation. TEER measurements of inserts without cells should be subtracted from the TEER values obtained with inserts containing cells. TEER values will be in Ω cm^2^.

**Days 4–6:**

1. Avoid acidification by refreshing the medium for NHA in the lower chamber and HBMEC inside the insert by gently aspiring the old medium and replacing it with the appropriate fresh medium.
2. Measure TEER twice each day.
3. Once the measured TEER value is near 200 ± 50 Ω cm^2^, there are sufficient tight junctions and interactions present between the HBMEC and NHA in the model. It should be ready for use.

### Validation of the model with transmigration assays

Chemotactic-cytokines chemokine receptor type 7 (CCR7) is one of the most prominent chemokine receptors in the adaptive immune system and an important migration receptor [18]. It is expressed in various subsets of immune cells [19], including monocytes [20]. The receptor has two specific ligands, C—C motif chemokine 19 (CCL19) and CCL21 [19]. These are up-regulated during neuro-inflammation [21], [22]. We used freshly isolated monocytes in CCR7-dependent transmigration assays to assess the co-culture of HBMEC and NHA model of cell migration through the BBB.

#### Materials and reagents

• Lymphocyte Separation Medium (Wisent, Cat# 305-010-CL).
• EasySep human monocytes enrichment kit without CD16 depletion (Stemcell Technologies, Cat# 19059, Vancouver, BC, Canada).
• Cell Dissociation Buffer (Life Technologies, Cat# 13151-014).
• Bovine Serum Albumins (BSA; Sigma–Aldrich, Cat# ALB001-100).
• CCL19 (R&D Systems, Cat# 361-MI-025, Minneapolis, MN, USA).
• Prostaglandin E2 (PGE2; Sigma–Aldrich, Cat# P5640-1MG).
• Antigen-presenting cells (APCs) from mouse anti-human cluster of differentiation 14 (CD14; BD Biosciences, Mississauga, ON, Canada, Cat# 340436).
• Mouse IgG2a APC (R&D Systems, Cat# MAB004).
• FACSCalibur Flow Cytometer System (BD Bio-sciences, San Jose, CA, USA).
• CellQuest software (BD Biosciences).

#### Procedure

1. Peripheral blood mononuclear cells (PBMCs) were isolated from healthy donors with Lymphocyte Separation Medium, according to the manufacturer's instructions.
2. Monocytes were isolated from PBMCs using EasySep human monocyte enrichment kit without cluster of differentiation 16 (CD16) depletion, according to the manufacturer's instructions. Magnetic immunoselection yielded an average purity of 98%. The purity was assessed by flow cytometric analyses, as recommended by the manufacturer, and isolated monocytes were stained with cluster of differentiation 14 (CD14)-fluorescein isothiocyanate (FITC) and anti-Biotin-phycoerythrin (PE) that labeled non-monocytes with a fluorescent dye.
3. Freshly isolated monocytes were maintained in culture in RPMI 1640 medium supplemented with 10% heat-inactivated fetal bovine serum (FBS) alone or with PGE2 that has been previously shown to up-regulate CCR7 expression in monocytes [20].
4. On the day of transmigration assays, cells were harvested using Cell Dissociation Buffer, as described by the manufacturer's instructions.

The BBB inserts were prepared as described earlier with the following modifications:

1. NHA medium was removed from the wells’ lower chambers and the new medium immediately supplied.
2. Either 400 μL of 300 ng mL^−1^ chemokine CCL19 solution diluted in NHA medium, or NHA medium alone, was added to wells’ lower chambers. The NHA medium was used by itself to control for spontaneous migration.
3. The HBMEC medium was gently removed from inserts and the new solution immediately supplied.
4. 1.5 × 10^5^ freshly isolated blood monocytes were resuspended in 150 μL NHA medium and then added to upper chambers.
5. The cells were incubated at 37 °C and 5% CO_2_ for 4 h.
6. 300-μL aliquots of the cells in the lower chamber were removed and washed twice in 3% BSA in PBS.
7. Cells were stained with APC mouse anti-human CD14 or with the corresponding isotype, APC mouse anti-human IgG2a. Both APCs were diluted 1:200 with 3% BSA in PBS.
8. CD14+ cells were counted with BD FACSCalibur by acquiring events for 60 s using CellQuest software. The percentage of cells that migrated was calculated as follows: the number of cells that migrated in response to medium only was subtracted from the number of cells that migrated in response to the medium supplemented with CCL19. This number was reported relative to the total number of cells.

#### Additional information

##### Validation of the BBB integrity

**Fig. 2:**
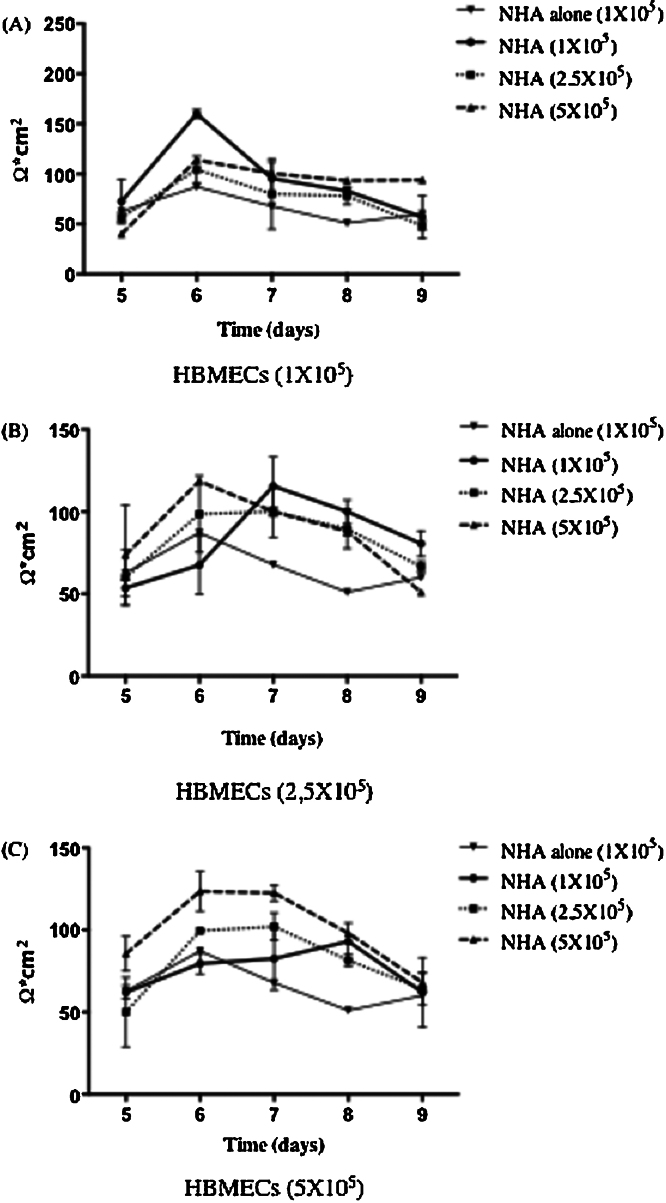
Time course of Transendothelial Electrical Resistance (TEER) of cells in the model. TEER was used to assess the formation of the BBB. (A) 1 × 10^5^ HBMEC, (B) 2.5 × 10^5^ HBMEC, and (C) 5 × 10^5^ HBMEC. Electrical resistance of the blank inserts with media alone was subtracted from the TEER of co-cultures. Data represent the mean of three different experiments, each with two TEER measurements, ±SD.

**Fig. 3:**
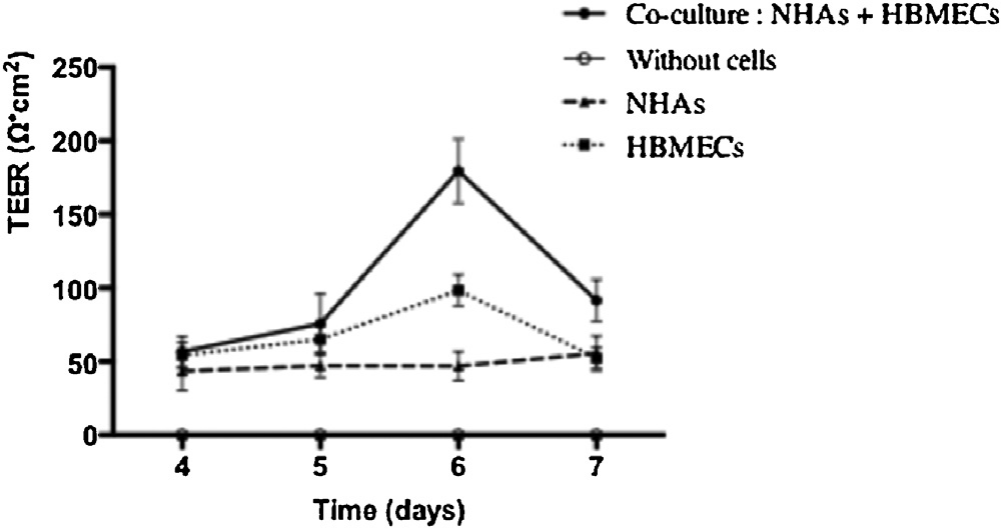
Development of TEER in different monolayers and co-cultivation cultures. The co-culture TEER values were evaluated by subtracting the TEER of the blank insert. Data represent the mean ± SD. Each co-culture model (*n*) was carried out in triplicate and TEER measurements were taken twice (day 6: *n* = 12, day 4, 5 and 7: *n* = 5).

To optimize the human BBB model, we performed a series of preliminary experiments to determine cell concentrations that lead to the formation of a functional BBB. To do so, different concentrations of HBMEC and NHA cells were tested. The functionality of the BBB was confirmed by determining the TEER value over 5 days at different concentrations of HBMEC and NHA (Fig. 2). Data suggest that 6 days of co-cultivation of HBMEC and NHA at a concentration of 1 × 10^5^ cells insert^−1^ produces the most successful BBB model, as determined by the TEER value (160 ± 30 Ω cm^2^). In addition, we validated the importance of NHA interactions with HBMEC in the formation of a tight BBB. TEER values of primary cells alone or combined were taken over 4 days (Fig. 3). HBMECs combined with NHA demonstrated the highest TEER, 180 ± 19 Ω cm^2^, while TEER values of cells alone were lower: values for HBMEC were 98 ± 10 Ω cm^2^; values for NHA were 47 ± 6 Ω cm^2^.

**Fig. 4:**
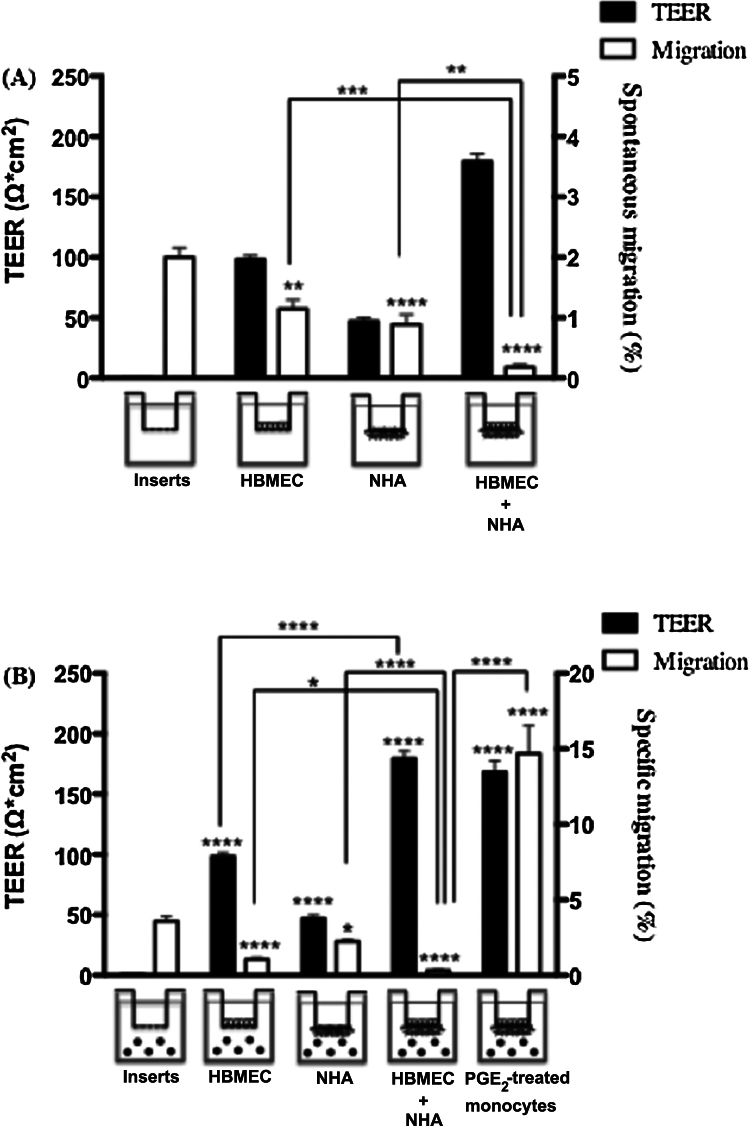
TEER and the percentage of blood monocytes that migrated across the BBB in different models. The cell-induced as well as co-culture TEER values were evaluated by subtracting the TEER of the blank insert. (A) Percentage of spontaneous migration vs. TEER values. (B) Percentage of specific migration toward CCL19 vs. TEER values. For the last condition, monocytes were treated for 24 h with PGE2 before the migration assay through the BBB model. Data represent the mean of four independent experiments, ±SD. Statistical analysis was performed using Student *t*-test; *p < 0.05; **p < 0.01, ***p < 0.001, ****p < 0.0001).

The permeability toward monocyte transmigration confirmed that the co-cultured endothelial monolayer was intact in our BBB model, consistent with a “tight” BBB (Fig. 4). The number of monocytes that spontaneously migrated was 0.18% in the co-cultures model, which was lower than through the filter alone, the HBMEC monolayer, or the NHA monolayer (2.01%, 1.14%, and 0.89%, respectively; Fig. 4A). We also determined the specific migration in response to CCL19 (Fig. 4B). Results confirmed that the formation of a tight BBB limits the migration of freshly isolated monocytes. Since we previously demonstrated that treatment with PGE2 increases CCR7 functionality in monocytes [20], PGE2-treated monocytes were tested in the BBB model. Up-regulation of CCR7 expression led to a better transmigration through the BBB model.

##### Overall utility and limitations of the BBB model

Altogether, our results suggest that the optimized in vitro human BBB model using HBMEC and NHA can serve as a reliable standard system for human monocyte transmigration assays in the central nervous system. In this co-culture model, TEER measurements were used to assess the BBB integrity. However, the presence of proteins characteristic of tight junctions was not evaluated by immunostaining. Instead, data obtained from spontaneous migration of monocytes in transmigration assays indirectly confirmed the BBB integrity of the proposed model.

## Conflict of interests

The authors declare that there are no conflicts of interest.

## References

[1] Rubin L.L., Staddon J.M. (1999). The cell biology of the blood–brain barrier. Annu. Rev. Neurosci..

[2] Cucullo L., Hossain M., Rapp E., Manders T., Marchi N., Janigro D. (2007). Development of a humanized in vitro blood–brain barrier model to screen for brain penetration of anti-epileptic drugs. Epilepsia.

[3] Persidsky Y., Stins M., Way D., Witte M.H., Weinand M., Kim K.S., Bock P., Gendelman H.E., Faila M. (1997). A model for monocyte migration through the blood–brain barrier during HIV-1 encephalitis. J. Immunol..

[4] Persidsky Y., Ghorpade A., Rasmussen J., Limoges J., Liu X.J., Stins M., Faila M., Kim K.S., Way D., Weinand M., Carhart L., Gendelman H.E. (1999). Microglial and astrocyte chemokines regulate monocyte migration through the blood–brain barrier in human immunodeficiency virus-1 encephalitis. Am. J. Pathol..

[5] Ifergan I., Kébir H., Bernard M., Wosik K., Dodelet-Devillers A., Cayrol R., Arbour N., Prat A. (2008). The blood–brain barrier induces differentiation of migrating monocytes into Th17-polarizing dendritic cells. Brain.

[6] Crone C., Christensen O. (1981). Electrical resistance of a capillary endothelium. J. Gen. Physiol..

[7] Hayashi K., Nakao S., Nakaoke R., Nakagawa S., Kitagawa N., Niwa M. (2004). Effects of hypoxia on endothelial/pericytic co-culture model of the blood–brain barrier. Regul. Pept..

[8] Garberg P., Ball M., Borg N., Cecchelli R., Fenart L., Hurst R.D., Lindmark T., Mabondzo A., Nilsson J.E., Raub T.J. (2005). In vitro models for the blood–brain barrier. Toxicol. In Vitro.

[9] Nakagawa S., Deli M.A., Kawaguchi H., Shimizudani T., Shimono T., Kittel A., Tanaka K., Niwa M. (2009). A new-blood–brain barrier model using primary rat brain endothelial cells, pericytes and astrocytes. Neurochem. Int..

[10] Nakagawa S., Deli M.A., Nakao S., Honda M., Hayashi K., Nakaoke R., Kataoka Y., Niwa M. (2007). Pericytes from brain microvessels strengthen the barrier integrity in primary cultures of rat endothelial cells. Cell. Mol. Neurobiol..

[11] Srinavasan B., Kolli A.R., Esch M.B., Abaci H.E., Shuler M.L., Hickman J.J. (2015). TEER measurement techniques for in vitro barrier model systems. J. Lab. Autom..

[12] Janzer R.C., Raff M.C. (1987). Astrocytes induce blood–brain barrier properties in endothelial cells. Nature.

[13] Deli M.A. (2007). Blood–brain Barrier Models. Handbook of Neurochemistry and Molecular Neurobiology.

[14] Kuo Y.C., Lu C.H. (2011). Effect of human astrocytes on the characteristics of human brain-microvascular endothelial cells in the blood–brain barrier. Colloids Surf.: Biointerfaces.

[15] Audus K., Borchardt R. (1986). Characterization of an in vitro blood–brain barrier model system for studying drug transport and metabolism. Pharm. Res..

[16] Dehouck M.P., Méresse S., Dehouck B., Fruchart J.C., Cecchelli R. (1992). In vitro reconstituted blood–brain barrier. J. Control. Release.

[17] Zucco F., Batto A.F., Bises G. (2005). An inter-laboratory study to evaluate the effects of medium composition on the differentiation and barrier function of Caco-2 cell lines. Altern. Lab. Anim..

[18] Schmid M.A., Takizawa H., Baumjohann D.R., Saito Y., Manz M.G. (2011). Bone marrow dendritic cell progenitors sense pathogens via Toll-like receptors and subsequently migrate to inflamed lymph nodes. Blood.

[19] Förster R., Davalos-Misslitz A.C., Rot A. (2008). CCR7 and its ligands: balancing immunity and tolerance. Nat. Rev. Immunol..

[20] Côté S.C., Pasvanis S., Bounou S., Dumais N. (2009). CCR7-specific migration to CCL19 and CCL21 is induced by PGE2 stimulation in human monocytes: involvement of EP2/EP4 receptors activation. Mol. Immunol..

[21] Krumbholz M., Theil D., Steinmeyer F., Cepok S., Hemmer B., Hofbauer M., farina C., Derfuss T., Junker A., Arzberger T., Sinicina I., Hartle C., Newcombe J., Hohlfeld R., Meinl E. (2007). CCL19 is constitutively expressed in the CNS, up-regulated in neuroinflammation, active and also inactive multiple sclerosis lesions. J. Neuroimmunol..

[22] Damas J.K., Landro L., Fevang B., Heggelund L., Froland S.S., Aukrust P.P. (2009). Enhanced levels of the CCR7 ligands CCL19 and CCL21 in HIV infection: correlation with viral load, disease progression and response to highly active antiretroviral therapy. AIDS.

